# In-Silico Identified New Natural Sortase A Inhibitors Disrupt *S. aureus* Biofilm Formation

**DOI:** 10.3390/ijms21228601

**Published:** 2020-11-14

**Authors:** Kishore Reddy Venkata Thappeta, Li Na Zhao, Choy Eng Nge, Sharon Crasta, Chung Yan Leong, Veronica Ng, Yoganathan Kanagasundaram, Hao Fan, Siew Bee Ng

**Affiliations:** 1 Singapore Institute of Food and Biotechnology Innovation (SIFBI), Agency for Science, Technology and Research (A*STAR), 31 Biopolis Way, #01-02 Nanos, Singapore 138669, Singapore; kishorertv@sifbi.a-star.edu.sg (K.R.V.T.); ngece@sifbi.a-star.edu.sg (C.E.N.); sharonc@sifbi.a-star.edu.sg (S.C.); leongcy@sifbi.a-star.edu.sg (C.Y.L.); ngwp@sifbi.a-star.edu.sg (V.N.); 2 Bioinformatics Institute (BII), Agency for Science, Technology and Research (A*STAR), 30 Biopolis Street, #07-01 Matrix, Singapore 138671, Singapore; zhaol@imcb.a-star.edu.sg; 3 Institute of Molecular and Cell Biology (IMCB), Agency for Science, Technology and Research (A*STAR), 61 Biopolis Drive, #3-09 Proteos, Singapore 138673, Singapore

**Keywords:** anti-biofilm activity, sortase A inhibitor, *Staphylococcus aureus*, MRSA, fibrinogen, molecular docking, virtual screening, natural products, skyrin

## Abstract

Sortase A (SrtA) is a membrane-associated enzyme that anchors surface-exposed proteins to the cell wall envelope of Gram-positive bacteria such as *Staphylococcus aureus*. As SrtA is essential for Gram-positive bacterial pathogenesis but dispensable for microbial growth or viability, SrtA is considered a favorable target for the enhancement of novel anti-infective drugs that aim to interfere with key bacterial virulence mechanisms, such as biofilm formation, without developing drug resistance. Here, we used virtual screening to search an in-house natural compound library and identified two natural compounds, N1287 (Skyrin) and N2576 ((4,5-dichloro-1H-pyrrol-2-yl)-[2,4-dihydroxy-3-(4-methyl-pentyl)-phenyl]-methanone) that inhibited the enzymatic activity of SrtA. These compounds also significantly reduced the growth of *S. aureus* but possessed moderate mammalian toxicity. Furthermore, *S. aureus* strains treated with these compounds exhibited reduction in adherence to host fibrinogen, as well as biofilm formation. Hence, these compounds may represent an anti-infective therapy without the side effects of antibiotics.

## 1. Introduction

Antibiotic resistance is a major global health threat to the effective combat of infections caused by bacteria. Most recent reviews on antibiotic resistance have specified an urgent need of antimicrobial arsenals to curb these superbugs, if not it might lead to the death of more than 10 million people per year by 2050, with a healthcare burden of 100 trillion USD [1,2]. To tackle this rising problem, it is important that novel treatments be developed to substitute the current broad-spectrum antibiotics, for example by selectively targeting and eliminating disease causing bacteria without altering the community organization of the neighboring microbiota [3,4,5,6]. These novel curatives contain both anti-virulence compounds that hinder bacterial pathogenesis and perseverance, along with novel bactericidal or bacteriostatic compounds that act towards very limited numbers of infective pathogens [3]. Several Gram-positive bacteria are prominently widespread and liable for deaths due to bacterial infections globally. *Staphylococcus aureus* is a primary human commensal and opportunistic Gram-positive pathogen and causes a wide variety of human diseases such as endocarditis, pneumonia, septic shock, necrotizing fasciitis, osteomyelitis and blood infections [7,8,9]. This pathogen is also one of the primary causes of both hospital and community-acquired infectious diseases [10]. The significance of *S. aureus* has been augmented due to the prevalence of multidrug-resistant (MDR) *S. aureus*, which causes deaths and currently is a medical challenge with limited therapeutic options [11,12]. *S. aureus* and other Gram-positive pathogenic bacteria interact with host cells and tissues via surface proteins and/or pili proteins which play a crucial role throughout the infection process. These surface proteins could also stick to the hosts or deliver some means for the bacteria to flee from the host’s immune response [13]. Therefore, an alternative potential way to treat infections produced by *S. aureus* and other Gram-positive pathogens is to target their surface proteins, which often work as virulence factors [14]. *S. aureus* and other Gram-positive bacteria use sortase enzymes to adhere surface proteins to their cell walls [15,16,17]. In *S. aureus*, 25 diverse LPXTG surface proteins known as MSCRAMMs (microbial surface components recognizing adhesive matrix molecules) are attached to the cell wall by the sortase A (SrtA) enzyme [18]. SrtA recognizes and cuts the LPXTG recognition design amongst the threonine (T) and glycine (G) residues of the surface proteins and consequently activates amide bond formation between the C-terminal end of the surface protein and the peptidoglycan cross-bridges of the cell wall [19,20]. SrtA mutants have deficits in the expression of surface proteins with different LPXTG motifs, leading to a reduced capability to infect the host [21]. Moreover, SrtA inhibition in *S. aureus* causes significant attenuation in its bacterial virulence, including binding activity to fibronectin, fibrinogen and lgG, as well as reduced stages of biofilm establishment in some *Staphylococcal* strains [22,23]. Since SrtA is not necessary for microbial growth and viability, inhibition of SrtA would be expected to enforce a reduced selection force for both the rise and extent of the resistance mechanism [24]. Therefore, SrtA has been well regarded as a vital target of anti-virulence drugs that are alternatives to broad-spectrum antibiotics [25,26,27].

Several approaches have been engaged to hunt for SrtA inhibitors. These approaches include high-throughput screening (HTS) of natural or synthetic compound libraries, virtual screening, and rational design of synthetic peptides and small molecules [23,24,25,26,27]. Natural products have been considered as an enduring source of novel drug leads. They have functional variability and are being conquered for an array of novel bactericidal or anti-virulence agents against bacterial infections [28,29]. In the present study, we performed virtual screening of compounds from the A*STAR Natural Organism Library (NOL) [29] to identify inhibitors of SrtA. Eleven compounds identified by virtual screening against the crystal structures of *S. aureus* SrtA were further evaluated by FRET enzyme assay. Two compounds, N1287 and N2576, displayed SrtA inhibition along with reduced adherence to fibrinogen as well as interfered with biofilm formation. These two compounds also possess anti-staphylococcus activity.

## 2. Results

### 2.1. Virtual Screening and Validation of SrtA Inhibitors

**Figure 1:**
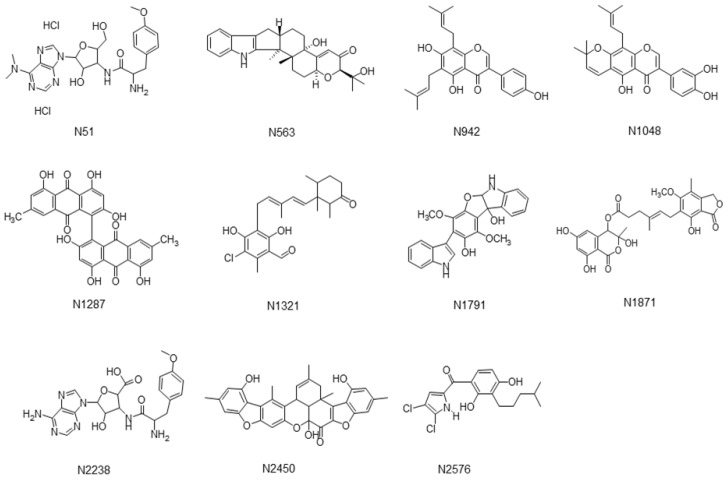
Structures of eleven in silico predicted natural compounds.

To be an inhibitor of sortase A (SrtA), the compound must have higher binding affinity towards the catalytic domain of this enzyme than the LPETG peptide. We therefore applied this principle in a virtual screening process to find potential inhibitors of SrtA in our Natural Organism Library (NOL). Firstly, the 2600 compounds derived from the NOL were docked to each of the three SrtA structures (PDB ID: 1T2P, 1T2W, and 1T2W_C184, see Materials and Methods) and ranked by their docking scores independently. Secondly, compounds were prioritized if their rankings were in the top 500 from at least two of the three docking runs (consensus hits), and also in the top 200 from at least one of the three docking runs. Finally, we manually selected eleven compounds from these consensus hits for experimental testing, taking into account their intermolecular hydrophobic interactions, and the number of polar atoms from the ligands that are satisfied/unsatisfied (Table 1) [30]. The chemical structures of these eleven ligand candidates are shown in Figure 1.

**Figure 2:**
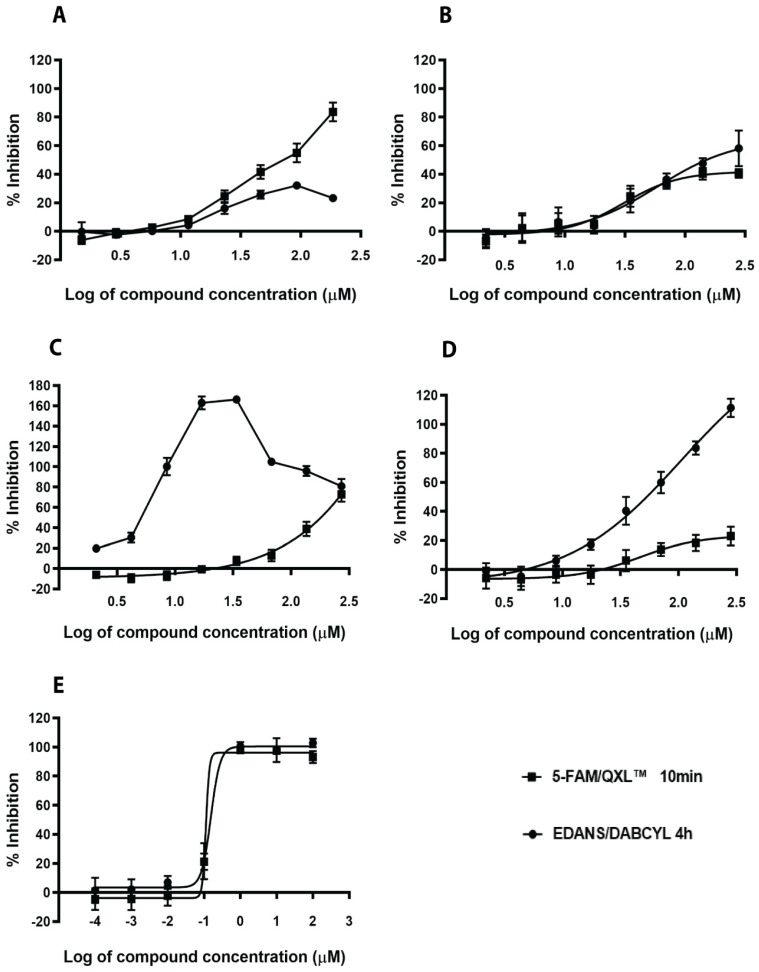
Dose response of hit compounds and known inhibitors on sortase A. Inhibition of Sortase A (SrtA) activity was measured at 10 min or 4 h using two sortase A substrates. N1287 (**A**), N2576 (**B**), curcumin (**C**), chlorogenic acid (**D**) and p-HMB (**E**). The results are mean values and SE of three replicates.

We evaluated these eleven in silico hits on their capability to hinder the enzymatic activity of *S. aureus* SrtA using commercially available enzymes and two different FRET substrates, DABCYL-LPETG-EDANS and 5-FAM/QXL (Table 2). The fluorescence of 5-FAM is less interfered by the autofluorescence of components in natural and investigation samples and thus acts as an activity confirmation assay. The 5-FAM/QXL FRET substrate is more susceptible to SrtA cleavage than the traditionally used DABCYL/EDANS substrate, and could detect activity of SrtA inhibitors within 10 min of substrate addition. On the other hand, the enzymatic reaction with the DABCYL/EDANS substrate required incubation for at least 4 h in order to achieve sufficient signal-to-background ratios. We also tested two reported natural inhibitors, curcumin and chlorogenic acid, and one synthetic inhibitor, p-hydroxymercuribenzoic acid (p-HMB), for activity comparison. The results are summarized in Table 2. Representative dose-responsive curves of two in silico hits and known inhibitors are shown in Figure 2. Two of the in silico hits, N1287 and N2576, inhibited the enzymatic activity of SrtA with IC_50_ less than 50 µM (Figure 2A,B). Curcumin exhibited autofluorescence (Figure 2C) but showed a stable dose response profile after 24 h incubation in the dark (Figure S1A) with an IC_50_ of 7.9 ± 1.0 µM and 23 ± 1.0 µM, respectively, for the DABCYL/EDANS and 5-FAM/QXL FRET substrates. The inhibitory activity of chlorogenic acid increased gradually with time for the FAM/QXL assay and showed moderate activity with IC_50_ of 103 ± 31 µM after 24 h of enzymatic reaction (Figure 2D and Figure S1B). N1287 and N2576 were found to be more active than chlorogenic acid. The IC_50_ values of the known SrtA inhibitor, p-hydroxymercuribenzoic acid, were within the reported range (Table 2 and Figure 2E).

### 2.2. Structure Analysis of SrtA-Ligand Complexes

**Figure 3:**
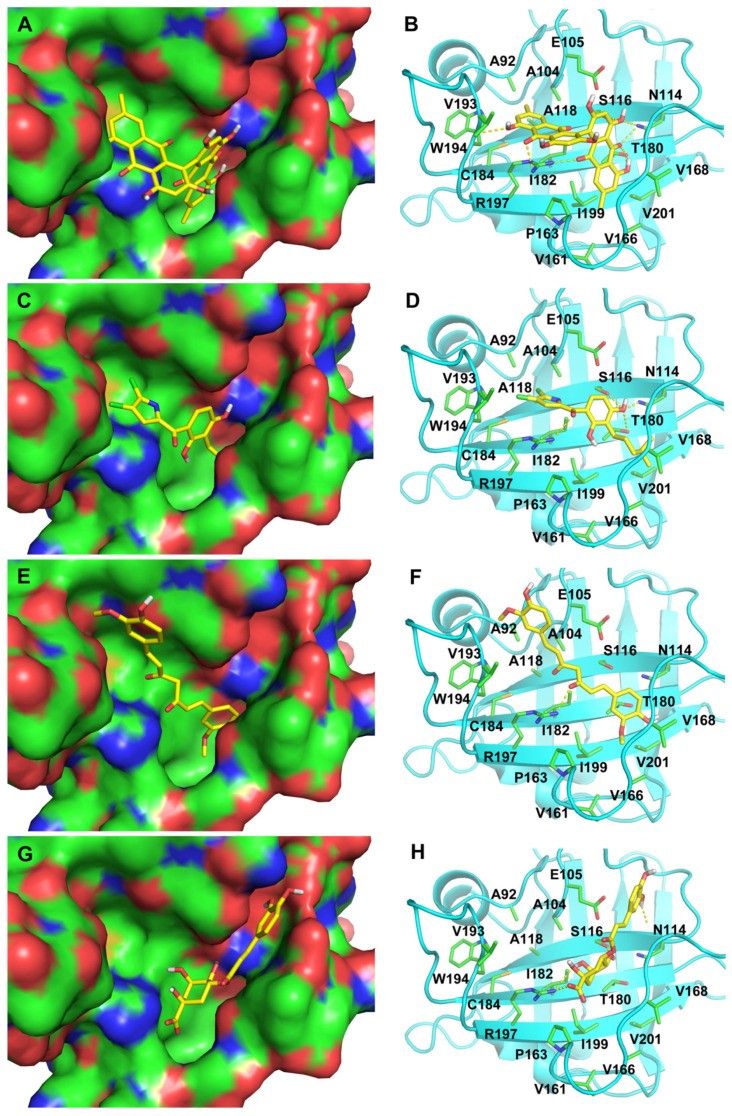
Docking poses of four compounds, namely (**A**,**B**), N1287, (**C**,**D**) N2576, (**E**,**F**), curcumin, and (**G**,**H**) chlorogenic acid. The compounds are shown as stick. The ligand binding site in the SrtA apo structure (PDB ID: 1T2P) is shown as surface in the panels on the left side (**A**,**C**,**E**,**G**), and as cartoon in the panels on the right side (**B**,**D**,**F**,**H**) with the sidechains of the binding residues highlighted as stick. These figures are generated by PyMOL [32].

In the apo structure of SrtA (PDBID 1T2P), a V-shaped pocket is formed by the β4, β7 and β8 strands on one side of the **β**-barrel, together with three surrounding loops. The left side of the pocket is a hydrophobic tunnel formed by Ala92, Ala104, Ala118, Val161, Pro163, Val166, Val 168, Ile182, Val193, Trp194, Ile199, and Val201, along with two putative catalytic residues Cys184 and Arg197 [31]. The right side of the pocket consists of several polar residues Glu105, Asn114, Ser116, and Thr180. The docking poses of the two confirmed SrtA inhibitors N1287 and N2576 are illustrated in Figure 3 (Figure 3A–D). The docking poses of two known SrtA inhibitors, curcumin and chlorogenic acid, are also provided for comparison (Figure 3E–H). All four inhibitors form a hydrogen bonding interaction or salt bridge with the guanidinium moiety of Arg197. N1287, N2576, and curcumin form extensive interactions with residues in the hydrophobic tunnel. In particular, the aromatic moiety from N1287 and N2576 forms a cation-π interaction with Arg197. N1287, N2576, and chlorogenic acid also form hydrogen bonding interactions with polar residues on the right side of the pocket, such as Asn114 and Ser116.

### 2.3. In Silico Hits Affect S. aureus Growth and Have Moderate Mammalian Toxicity

**Figure 4:**
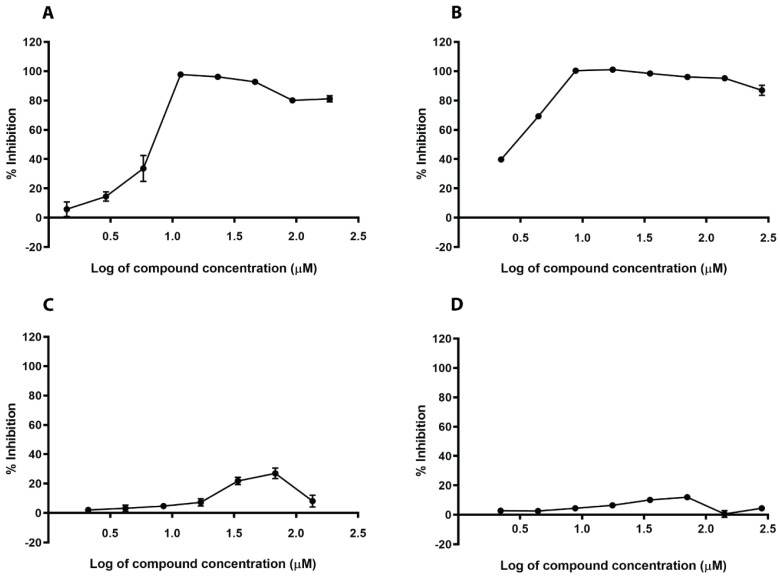
Effect of hit compounds and known inhibitors on the growth of *S. aureus* Newman strain. N1287 (**A**), N2576 (**B**), curcumin (**C**), and chlorogenic acid (**D**). The results are mean values and SE of three replicates.

**Figure 5:**
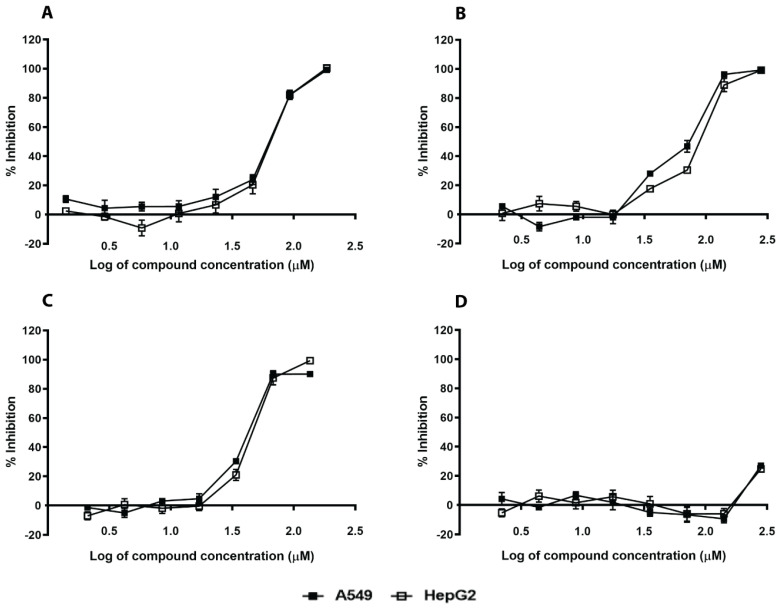
Cytotoxicity of hit compounds and known inhibitors against A549 (▪) and HepG2 (□) cancer cell lines. N1287 (**A**), N2576 (**B**), curcumin (**C**), and chlorogenic acid (**D**). The results are mean values and SE of three replicates.

We assessed the growth inhibitory activity of both N1287 and N2576 on *S. aureus* using the modified microbroth dilution assay. Both compounds inhibited the growth of *S. aureus* with a minimum inhibitory concentration (MIC) of 5–8 µM (Table S2; Figure 4 and Figure S2). These two compounds also inhibited the growth of methicillin resistant *S. aureus* (MRSA) with an MIC of 25–80 µM (Table S1 and Figure S3). However, the two reported SrtA inhibitors, curcumin and chlorogenic acid, did not inhibit the growth of *S. aureus* at 200 µM. Both N1287 and N2576 also displayed moderate toxicity (IC_50_ 60–90 µM) against A549 and HepG2 cells (Table S1; Figure 5).

### 2.4. In Silico Hits Reduce Biofilm Formation

**Figure 6:**
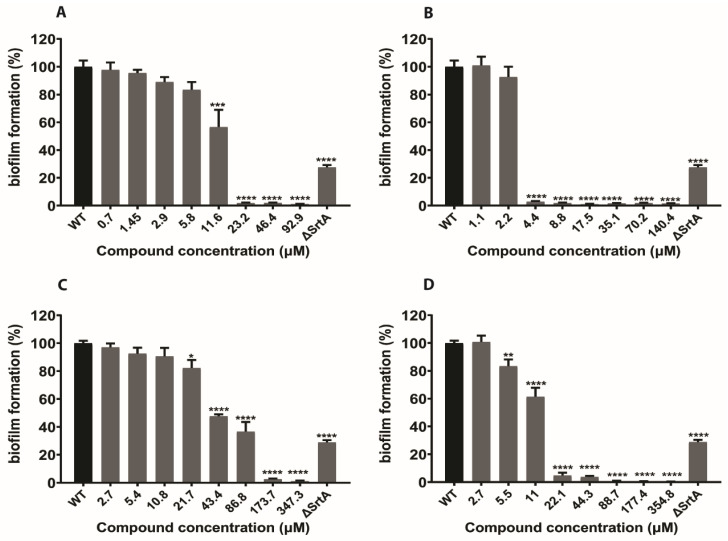
Effect of the known inhibitors of SrtA and selected hits on *S. aureus* (strain Newman) biofilm formation. Inhibition of *S. aureus* biofilm formation was determined using CV assay. N1287 (**A**), N2576 (**B**), curcumin (**C**) and p-HMB (**D**). The results are presented as the mean percentage of biofilm formation ± S.E of six replicates (two independent experiments) relative to the untreated control (wild type). * *p* < 0.05, ** *p* < 0.01, *** *p* < 0.001 and **** *p* < 0.0001.

**Figure 7:**
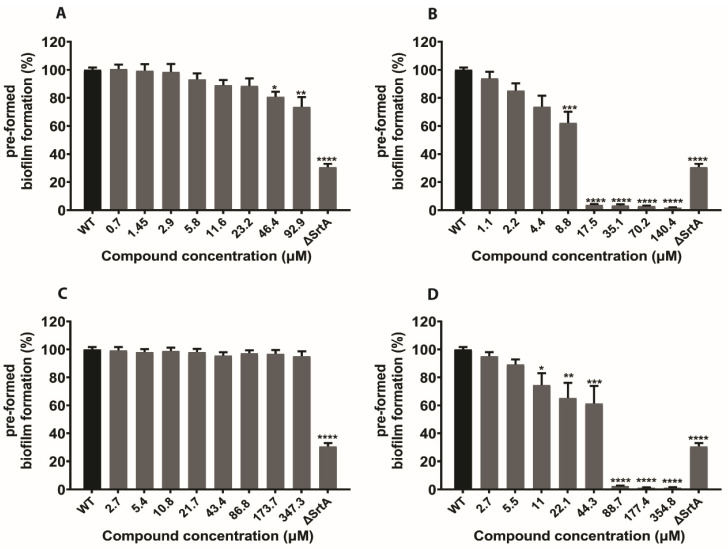
Effect of the known inhibitors of SrtA and selected hits on pre-formed biofilms of *S. aureus* cells (strain Newman). Inhibition of *S. aureus* pre-formed biofilm formation was determined using crystal violet staining (CV) assay. N1287 (**A**), N2576 (**B**), curcumin (**C**) and p-HMB (**D**). The results are presented as the mean percentage of biofilm formation ± S.E of six replicates (two independent experiments) relative to the untreated control (wild type). * *p* < 0.05, ** *p* < 0.01, *** *p* < 0.001 and **** *p* < 0.0001.

Bacteria develop their ability to fight antibiotics and other antimicrobial agents by establishing biofilms [33,34,35]. Previous studies revealed that SrtA played a significant role in biofilm formation. To determine whether our natural SrtA inhibitors N1287 and N2576 hindered biofilm formation, *S. aureus* biofilm formation was determined by a crystal violet staining (CV) assay, with or without these two compounds. Natural inhibitor curcumin and synthetic inhibitor p-HMB were included for comparative study. As anticipated, the results showed that *S. aureus* biofilm formation was inhibited by all four compounds. Quantitative investigation showed that N1287 and N2576 inhibited biofilm formation by >90% at concentrations 23 and 4.4 µM, respectively (Figure 6A,B). Moreover, N2576 demonstrated >90% inhibition on preformed biofilms at 17 µM (Figure 7B). IC_50_ (concentration at 50% inhibition) values of 2.9 and 10 µM correspond to the inhibitory effect of N2576 on biofilm formation as well as pre-formed biofilms, respectively. The IC_50_ of N1287 on *S. aureus* biofilm formation is 12 µM (Table S1). The known SrtA inhibitors, curcumin and p-HMB showed >90% inhibition on biofilm formation at the concentrations of 174 and 22 µM, respectively (Figure 6C,D), and IC_50_ values of, respectively, 51 and 12 µM (Table S1). Furthermore, we evaluated the biofilm inhibitory activity of N1287 and N2576 on drug resistant *S. aureus* (MRSA). N2576 displayed >90% inhibition on MRSA biofilm formation and preformed biofilms (Figures S4B and S5) at 70 and 280 µM, respectively, while N1287 exhibited inhibitory activity on MRSA biofilm formation but not against pre-formed biofilms (Figures S4A and S5). Both N1287 and N2576 displayed better biofilm inhibitory activity compared to known inhibitors. Compound N2576 is more effective than N1287, p-HMB and curcumin.

Furthermore, we evaluated the anti-biofilm activity by another method using PrestoBlue cell viability reagent. The results demonstrated that the concentrations that showed a >90% reduction in the viability of the biofilm, were nearly the same as the concentrations that caused the same effect in the CV assay (Figure S6). However, compounds N1287 and N2576 showed a greater inhibitory effect on the growth of *S. aureus* Newman strain, with MIC values less than the SrtA IC_50_ values (Table S1 and Table 1). In both methods these two compounds displayed better anti-biofilm activity against *S. aureus.* Hence, the compounds N1287 and N2576 disturbed the biofilm structure by inhibiting the growth, as well as activity, of SrtA of *S. aureus.* We further examined whether the two compounds display any effect on the biofilm formation of Gram-negative bacterium and found that these two compounds (N1287 and N2576) did not show any effect on *E. coli* ATCC 25,922 biofilm formation (Figure S7). Similar results were observed with known SrtA inhibitors curcumin and p-HMB on *E. coli* biofilm formation (Figure S7). Therefore, these two compounds exhibited a biofilm inhibitory effect only on Gram-positive bacterial biofilms (*S. aureus*).

### 2.5. In Silico Hits Reduce the Adherence of S. aureus to Fibrinogen

**Figure 8:**
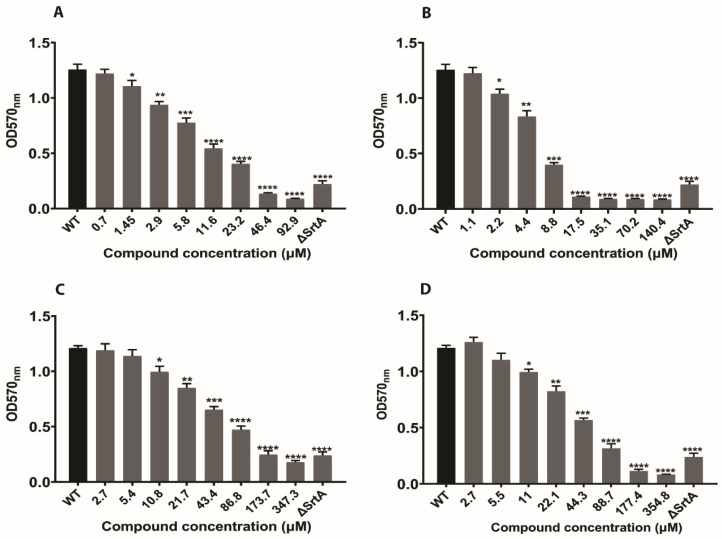
Effects of SrtA inhibitors on the adherence of *S. aureus* cells (strain Newman) to fibrinogen protein. N1287 (**A**), N2576 (**B**), curcumin (**C**), and p-HMB (**D**). The results are presented as the mean absorbance at 570 nm ± S.E of three replicates (two independent experiments) relative to the untreated control (wild type). * *p* < 0.05, ** *p* < 0.01, *** *p* < 0.001 and **** *p* < 0.0001.

Several different surface proteins in *S. aureus* are covalently attached to the cell wall by the transpeptidation reaction catalyzed by sortase A [36,37]. Surface proteins such as fibrinogen binding protein, fibronectin-binding proteins, clumping factors and ClfB are vital for the pathogenesis of *S. aureus*. Mutants that lack SrtA activity were futile in making surface proteins and are faulty in the establishment of infections [21]. To assess whether our SrtA inhibitors have a similar impact on *S. aureus* surface protein formation, we conducted a fibrinogen-binding assay which quantified the adherence of *S. aureus* cells to fibrinogen coated plates. The amount of *S. aureus* cells bound to the fibrinogen coated plates was determined by absorbance measurement after staining with crystal violet. The results revealed a significant reduction in the binding of *S. aureus* to fibrinogen. The compounds N1287 and N2576 displayed ≥90% prevention of *S. aureus* Newman adherence to fibrinogen at 46 and 17 µM, respectively (Figure 8A,B), compared to untreated wildtype strain. The known SrtA inhibitors curcumin and p-HMB exhibited a ≥90% reduction in adhesion to fibrinogen binding protein at >170 µM (Figure 8C,D). As expected, the Δ*SrtA* strain had reduced (30–35%) adhesion to fibrinogen due to the loss of its fibrinogen-binding activity (Figure 8).

## 3. Discussion

The rise and widespread occurrence of multiple-drug-resistant *S. aureus* (MRSA) represents a huge medical challenge in the treatment of clinical *S. aureus* infections [38,39]. Therefore, it is essential to develop alternative strategies to combat *S. aureus* infections. Probing new virulence inhibitors is one of the alternative strategies to treat infections initiated by multidrug resistant bacteria [40]. *S. aureus* uses the sortase A (SrtA) enzyme to display cell surface virulence factors involved in the establishment of various infections [22,23]. Hence, in the present study we searched for SrtA inhibitors from natural products as an alternative approach for treating *S. aureus* infections.

Natural products are of great variety with chemical, as well as biological diversity; they are a key source of therapeutic agents for infectious diseases [28,29]. Computational approaches have been facilitating the investigation of natural products to accelerate the early stage of drug discovery by efficiently identifying new leads and repurposing known natural ligands [41,42]. In the present study, we applied the structure-guided virtual screening approach to a database of natural products that are available in our Natural Organism Library [29]. In particular, we used an ensemble docking strategy, targeting multiple structures of sortase A (SrtA) and selecting consensus hits for experimental testing. Using this approach, we successfully identified two potential SrtA inhibitors N1287 and N2576 that exhibited SrtA inhibitory activity against *S. aureus* with an IC_50_ between 5.8–7.3 μM. N1287 (Skyrin) is a fungal metabolite characterized by a bisanthraquinone structure with two trihydroxyanthraquinone molecules (emodin) connected by a single C-C bond at position four. It was reported to be a promising drug candidate for cancer and diabetes treatment [43,44]. The docking pose of N1287 shows that the two anthraquinone moieties are T-shape stacked and occupy the majority of the V-shape ligand binding pocket, thus explained skyrin’s potency against SrtA (Figure 3A,B). Pyrrolomycins are a family of potent natural product antibiotics active against Gram-positive bacteria, yet with an elusive mechanism of action [45]. In a recent study, a number of pyrrolomycins were shown to have inhibitory activity toward SrtA, possessing IC_50_ values ranging from 130 to 300 μM [46]. In comparison to these reported pyrrolomycins, our analogue N2576 has an isohexane tail added on the phenyl ring. This modification allows N2576 to form a hydrophobic cluster with several surrounding valine residues (Figure 3C,D) in the catalytic domain of SrtA, and probably contributed to the observed enhanced potency. Our finding therefore provided a possible direction for future optimization of pyrrolomycin.

We also found that these two natural compounds reduced the biofilm formation, as well as biofilm viability (Figure 6 and Figure 7 and Figures S4–S6), and decreased the adhesion of *S. aureus* to fibrinogen (Figure 8). Additionally, these two compounds reduced MRSA biofilm formation (Figures S4 and S5). As expected, these two compounds did not show any inhibitory effect on Gram-negative bacterial (*E. coli* ATCC 25922) biofilms (Figure S7), which lack transpeptidase SrtA. Some earlier reports revealed that highly selective SrtA inhibitors do not cease bacterial growth and viability [21]. Very interestingly, these two SrtA inhibitors N1287 and N2576 exhibited antibacterial, as well as cytotoxic activity (Table S1). Furthermore, N2576 showed significant inhibition of the pre-formed biofilm formation. Previous studies on natural and synthetic pyrrolomycin analogues against *S. aureus* strains demonstrated anti-staphylococcal activity with a minimum inhibitory concentration (MIC) ranging from 0.004 to 25 μM [46,47]. The compounds N2576 and N1287 displayed potent anti-staphylococcal activity against *S. aureus* strains (ATCC 25,923 and Newman) with MICs between 5 and 8 μM, whereas they are less active against MRSA with MICs between at 25 and 80 μM in the present study. However, the MIC values of N1287 and N2576 were lower than their IC_50_ values for SrtA, suggesting that these two compounds inactivate additional processes important for cell viability apart from SrtA inhibition. Similarly, some antibacterial compounds with potent MIC values were reported to show SrtA inhibition [48,49,50,51].

Our results have revealed the potential of these two natural compounds N1287 and N2576 as anti-proliferation—as well as anti-biofilm—agents for treating *S. aureus* infection. Further mode-of-action studies will be necessary to determine the mechanism of inhibitory effect of these two compounds on bacterial growth. However, these two SrtA ligands (compounds), could be further optimized to achieve higher specificity and lower toxicity, and then used as lead compounds for drug development.

## 4. Materials and Methods

### 4.1. Reagents, Bacterial Strains and Cell Lines

Recombinant *S. aureus* sortase A (SrtA) and the two SrtA FRET substrates; DABCYL-Leu-Pro-Glu-Thr-Gly-EDANS (Bacterial Sortase Substrate I) and 5-FAM/QXL^™^ 520 sortase substrate were purchased from AnaSpec (Fremont, CA, USA). The standard inhibitor compounds: p-hydroxymercuribenzoic acid, gentamicin, puromycin, curcumin and chlorogenic acid were obtained from Sigma-Aldrich (St. Louis, MO, USA). Other chemicals were acquired from Sigma-Aldrich unless otherwise specified.

Reference bacterial strain *Staphylococcus aureus subsp. aureus* ATCC 25923, ATCC 33591 (MRSA), *E. coli* ATCC 25922, human lung carcinoma cells A549 ATCC CCL185 and human liver carcinoma cells HepG2 ATCC HB8065 were obtained from American Type Culture Collection (Manassas, VA, USA). *Staphylococcus aureus* Newman and its Δ*SrtA*-Newman strain were supplied by Professor Simon Foster as a gift. They were cultured at 37 °C in Tryptic soya broth (TSB) which was supplemented with erythromycin at 5µg/mL whenever necessary. Cation-adjusted Mueller Hinton (BD BBL, Sparks, MD, USA) broth and agar were used as growth media for the other *S. aureus*. A549 cells were cultivated in Dulbecco’s modified Eagle Medium (Gibco, Thermo Fisher Scientific, Waltham, MA, USA) and HepG2 cells were grown in minimum essential medium Eagle (Gibco, Thermo Fisher Scientific, Waltham, MA, USA) with Earle’s balanced salts and non-essential amino acids. Both media were supplemented with 4 mM l-glutamine, 2 mM sodium pyruvate, 10% fetal bovine serum, 100 U/mL penicillin and 100 µg/mL streptomycin. Both cell cultures were used between passages 18 and 20.

### 4.2. Chemical Compounds

The 11 selected compounds used for evaluation of the virtual screen results were isolated in-house and deposited in the NOL compound library [29], stored at −20 °C. The pureness of the 11 compounds were further confirmed by ^1^H NMR analysis (purity greater >90%) before submitted for assays testing.

### 4.3. Virtual Screening

The 3D structures of SrtA used for docking were taken from Protein Data Bank (PDB) (PDB ID: 1T2P and 1T2W) [31]. 1T2P and 1T2W are crystal structures of *S. aureus* (SrtA) determined at 2.1 Å and 1.8 Å resolution, respectively. 1T2P is a ligand-free (apo) structure while 1T2W is in complex with a peptide ligand with the sequence LPETG. The A chain from 1T2P and 1T2W was independently used as the receptor structure and prepared for docking using Dock 3.6 [52]. For 1T2W, the binding pocket used in docking was determined by the bound peptide. For 1T2P, we superposed it on 1T2W and employed the same binding pocket as that in 1T2W. Because 1T2W has one mutation C184A, we mutated residue Ala-184 back to wildtype Cys and optimized neighboring residues with SCWRL4 (1T2W_C184) [53]. In total three SrtA structures (1T2P, 1T2W, 1T2W_C184) were used in the virtual screening of 2521 compounds from NOL [29]. The docked compounds were categorized by the docking energy function, that is the sum of van der Waals, Poisson–Boltzmann electrostatic, and ligand desolvation penalty terms [54].

### 4.4. SrtA Enzyme Inhibition Assay

The effect of in silico hits on the enzymatic activity of *S. aureus* SrtA (AnaSpec AS-72229) was determined using two different FRET substrates, DABCYL-LPETG-EDANS (AnaSpec, San Jose, CA, USA) and 5-FAM/QXL^TM^ 520 (AnaSpec, San Jose, CA, USA. The latter substrate is a novel internally quenched fluorogenic peptide and the enzymatic activity of SrtA results in the release of 5-FAM fluorescence, which can be observed at Ex/Em = 490 nm/520 nm. The extended wavelength fluorescence of 5-FAM is less affected by the autofluorescence components in biological and test samples.

The enzymatic reactions were carried out in black, low-binding polystyrene 384 well plates (Greiner Bio-One). Test compounds were assayed in triplicate as an 8-point, 2-fold dilution series starting from concentrations where no precipitation could be observed. SrtA was pre-incubated in the reaction buffer with compounds for 1 h at room temperature prior to the addition of the FRET substrate. Assays were performed in 50 µL reactions with 50 ng of *S. aureus* sortase A and 25 µM of DABCYL-LPETG-EDANS substrate or 1 µM of 5-FAM/QXL^TM^ 520 substrate. The inhibitory activity of the compounds was determined by monitoring the rise in fluorescence intensity over 24 h at Ex/Em = 336 nm/490 nm for the DABCYL/EDANS substrate and Ex/Em = 490 nm/520 nm for the 5-FAM/QXL 520 substrate on a Tecan Infinite M1000 Pro reader. Compounds were tested in the absence of fluorophore to measure compound autofluorescence since strongly autofluorescent compounds may give false results. The synthetic compound, p-hydroxymercuribenzoic acid and natural sortase inhibitors, curcumin and chlorogenic acid were used as reference inhibitors.

Raw fluorescence data were corrected for autofluorescence before computing percentage inhibition values. These percentage inhibition values were plotted against compound concentration using GraphPad Prism version 8.0 by nonlinear regression analysis using least squares fit method for IC_50_ determination. Testing was performed twice to ensure reproducibility of results.

### 4.5. S. aureus Whole Cell Assay

Minimum inhibitory concentration (MIC) tests were performed according to procedures of the Clinical Laboratory Standards Institute (CLSI) with minor changes using the microbroth dilution technique in a 384-well setup. Inoculum of each *S. aureus* strain was prepared by suspending segregated colonies in sterile saline to a 0.5 McFarland standard. An inoculum of 2–8 × 10^5^ CFU/mL was incubated with the compounds for 24 h at 37 °C. Gentamicin (data not shown) was incubated in the same manner as the test compounds and served as a positive control. The effect of the compounds on bacterial growth was calculated by quantifying the optical density at 600 nm on on a microplate reader (Tecan, Infinite M1000, Salzburg, Austria). Dilutions of the inoculum were cultivated on agar to verify the validity of the inoculum seeding density and absence of contamination. All tests were executed in triplicate during two separate experiments.

### 4.6. Biofilm Inhibitory Assay

The effectiveness of SrtA inhibitors in reducing biofilm formation and growth was assessed using the crystal violet (CV) biofilm assay with the following modifications [55]. TSB with 2% glucose (TSB without glucose for *E. coli*) was inoculated with 10^5^ CFU/mL of *S. aureus* and *E. coli* overnight cultures. A total of 90µl of this cell suspension was then dispensed into 96-well microtiter plates containing 10 µL of different concentrations of the SrtA inhibitors. After 24 h growth at 37 °C the plates were washed with PBS to remove unbound cells and heat fixed at 60 °C for 30 min. Next, the wells were stained with 0.1% crystal violet solution for 15 min at room temperature. At the completion of the incubation plates were washed 3 times with PBS and dried. Then the crystal violet dye from the biofilm was solubilized with 200 µL of DMSO. A total of 100 µL of this solution was then moved to a new microtiter plate for absorbance measurement at 595 nm. For pre-formed biofilm assay, the plates were prepared and incubated as mentioned earlier, without SrtA inhibitors. After 24 h incubation, the culture supernatant was removed gently and biofilm carefully washed with sterile PBS. Next, 10 µL of different concentrations of SrtA inhibitors with 90 µL of TSB with 2% glucose were added carefully into the wells and incubated at 37 °C for 24 h. The amount of crystal violet staining of biofilm was measured as described above. The results were reported as percent inhibition normalized to the wild type control. Two independent experiments were performed in triplicate to ensure reproducibility.

### 4.7. Assessment of Biofilms Metabolic Activity

For this assay, 96-well microtiter plate was prepared as specified in the above biofilm assay. This assay was performed with the following modifications [56]. After 24 h incubation, culture supernatant was removed carefully and wells washed with sterile PBS. The metabolic activity of the biofilms was determined by adding 1:10 dilution of the PrestoBlue cell viability reagent (Life Technologies, Carlsbad, CA, USA) in PBS and incubated for 30 min at 37 °C. After incubation, the fluorescence was studied at 560 nm and 590 nm (excitation and emission, respectively) in a microplate reader. For pre-formed biofilm assay, the plates were prepared and incubated as mentioned earlier and the metabolic activity measured as described above. The outcome was defined as percent inhibition normalized to the wild type. Two independent experiments were performed in triplicate to ensure reproducibility.

### 4.8. Fibrinogen Binding Assay

Fibrinogen binding assay was completed as described but with slight revision [57]. A flat-bottom 96-well plate was covered with fibrinogen protein by adding 50 µL of 20 µg/mL of fibrinogen protein from bovine plasma diluted in saline and incubated at 4 °C for 24 h. The coated plates were gently rinsed twice with PBS and blocked for 60 min with 4% bovine serum albumin (BSA). The plates were then washed 3 times with PBS. For this assay, *S. aureus* Newman strain was cultivated in TSB at 37 °C to exponential phase (OD600 = 0.5) and then the bacterial culture was incubated with various concentrations of test compound or control for 2.5 h. Then the cells were pelleted by centrifugation at 10,000× *g* for 10 min, the supernatants were removed and the cell pellets stored at −20 °C. The following day, cell pellets were suspended in PBS and dispersed in 100 μL aliquots to distinct wells of fibrinogen-coated 96-well plates. After 2 h of growth at 37 °C, the bacterial supernatant was discarded and the wells were rinsed gently with 150 µl PBS. Then the bacterial cells were fixed with 2% (*v*/*v*) glutaraldehyde and incubated for 30 min at 25 °C. After a wash with PBS, bound cells were stained with 0.1% crystal violet dye for 15 min. Plates were rinsed twice again with PBS, enclosed with aluminum foil, then stored at room temperature to dry overnight. On the next day absorbance was measured at 570 nm. The results were described as the percent of adhesion inhibition normalized to the wild type control. Two independent experiments were performed in triplicate to ensure reproducibility.

### 4.9. Mammalian Cell Cytotoxicity Assay

A549 and HepG2 cells were seeded at 1500 and 2500 cells per well, respectively, in 384-well microplates and incubated at 37 °C in the presence of 5% CO_2_. The next day, cells were treated with compounds then incubated for an additional 72 h. To assess the cytotoxic effect of the compounds PrestoBlue cell viability reagent (Life Technologies, Carlsbad, CA, USA) was used. After the 72 h incubation with compounds, the 384-well assay plates were incubated with this viability indicator dye for 2 h (A549) and 4 h (HepG2) before fluorescence reading at Ex/Em = 560 nm/590 nm on the Tecan Infinite M1000 Pro reader. Puromycin (data not shown) was used as the positive control against both cell lines. All tests were executed in triplicate on two separate experiments.

### 4.10. Statistical Analysis

All graphs were constructed using GraphPad PRISM 8.0. Comparisons were done using one-way ANOVA. *p* < 0.05 was considered to be statistically significant.

## Figures and Tables

**Table 1:** Summary of the eleven natural products picked for experimental validation, comparing to curcumin and chlorogenic acid.

Compounds	Docking Scores/Ranks from 3 Different Sortase A Structures					
	1T2P		1T2W		1T2W_C184	
	Score	Rank	Score	Rank	Score	Rank
N51	−44.39	41	−39.14	97	−36.86	139
N563	−35.85	410	−37.95	141	−36.83	143
N942	−40.61	142	−41.59	33	−42.19	11
N1048	−36.9	330	−38.10	135	−39.76	4
N1287	−41.87	88	−36.01	322	N/A	N/A
N1321	−38.63	233	−37.60	158	−38.15	85
N1791	−40.29	164	−35.15	372	−36.33	176
N1871	−46.58	12	−40.03	67	−40.31	35
N2238	−45.30	25	−45.93	8	−50.42	2
N2450	−38.36	251	−39.76	76	−42.81	9
N2576	−34.61	464	−33.88	435	−36.65	156
Curcumin	−47.53	10	−35.85	339	−31.24	491
Chlorogenic acid	−28.19	>500	−32.24	>500	−28.19	>500

N/A—No docking results were generated when N1287 was docked to 1T2W_C184.

**Table 2:** Mean sortase A IC_50_ values of eleven in silico hits, two reported natural inhibitors (curcumin and chlorogenic acid), and a synthetic inhibitor p-hydroxymercuribenzoic acid. IC_50_ denotes the half maximal inhibitory concentration of the compound, as determined from GraphPad Prism software.

Compound ID	Compound Name	Sortase A IC_50_ (µM) (DABCYL-LPETG-EDANS Substrate; 4 h)	Sortase A IC_50_ (µM) (5-FAM/QXL Substrate; 10 min)
N51	Puromycin hydrochloride	>200	>200
N563	Paxilline	>200	>200
N942	6,8-diprenylgenistein	>200	>200
N1048	Auriculasin	>200	>200
N1287	Skyrin	24 ± 2.1	31 ± 0.3
N1321	Ascochlorin	>200	>200
N1791	Demethyl asterriquinone	>200	>200
N1871	Antibiotic F 13459	>200	>200
N2238	Chryscandin	>200	>200
N2450	Asticolorin B	>200	>200
N2576	(4,5-dichloro-1*H*-pyrrol-2-yl)-[2,4-dihydroxy-3-(4-methyl-pentyl)-phenyl]-methanone	47 ± 4.2	32 ± 6.3
	Curcumin	7.0 ± 1.8	107 ± 16
	Chlorogenic acid	88 ± 5.6	144 ± 16
	p-hydroxymercuribenzoic acid (p-HMB)	0.20 ± 0.07	0.18 ± 0.08

## Supplementary Materials

The following dose-responses data are available online at https://www.mdpi.com/1422-0067/21/22/8601/s1, Figure S1: Dose response of known inhibitors, curcumin (A) and chlorogenic acid (B), on sortase A. Inhibition of SrtA activity was measured at 24 h using two SrtA substrates. The results are mean values and SE of three replicates. Figure S2: Effect of hit compounds and known inhibitors on the growth of *S. aureus* ATCC 25923: N1287 (A), N2576 (B), curcumin (C), and chlorogenic acid (D). The results are mean values and SE of three replicates. Figure S3: Effect of hit compounds and known inhibitors on the growth of *S. aureus* ATCC 33591 (MRSA): N1287 (A), N2576 (B), curcumin (C), and p-HMB (D). The results are mean values and SE of three replicates. Figure S4. Effect of the known inhibitors of SrtA and selected hits on *S. aureus* ATCC 33591 (MRSA) biofilm formation and pre-formed biofilms. Inhibition of S. aureus biofilm formation was determined using CV assay. N1287 (A), N2576 (B), curcumin (C) and p-HMB (D). Figure S5: Crystal violet assay to assess the antibiofilm activity of selected hits and known inhibitors of SrtA on *S. aureus* ATCC 33591 (MRSA) biofilm formation (A) and pre-formed biofilms (B). Figure S6: Dose-response of the known inhibitors of SrtA and selected hits on S. aureus ATCC 33591 (MRSA) biofilm formation and pre-formed biofilms. Inhibition of S. aureus biofilm was determined using PrestoBlue cell viability reagent. N1287 (A), N2576 (B), curcumin (C), and p-HMB (D). Figure S7: Effect of the known inhibitors of SrtA and selected hits on *E. coli* ATCC 25922 biofilm formation. Inhibition of E. coli ATCC 25922 biofilm formation was determined using CV assay. N1287 (A), N2576 (B), curcumin (C) and p-HMB (D). Table S1: Biological activity of selected hits and standard inhibitors.

## Abbreviations

BSA	bovine serum albumin
CFU	colony-forming unit
CV	Crystal violet
DMSO	dimethyl sulfoxide
FRET	fluorescence resonance energy transfer
p-HMB	p-hydroxymercuribenzoic acid
IC_50_	half maximal inhibitory concentration
MDR	multidrug-resistant
MIC	minimum inhibitory concentration
PBS	Phosphate buffer saline
SrtA	sortase A
TSB	Tryptic soya broth

## References

[1] Piddock L.J.V. (2016). Reflecting on the final report of the O’Neill Review on Antimicrobial Resistance. Lancet Infect. Dis..

[2] Adeyi O., Baris E., Jonas O., Irwin A., Berthe F., Le Gall F., Marquez P., Nikolic I., Plante C., Schneidman M. (2017). Drug-Resistant Infections: A Threat to Our Economic Future.

[3] Spaulding C.N., Klein R.D., Schreiber H.L., Janetka J.W., Hultgren S.J. (2018). Precision antimicrobial therapeutics: The path of least resistance?. NPJ Biofilms Microbiomes.

[4] Parrino B., Schillaci D., Carnevale I., Giovannetti E., Diana P., Cirrincione G., Cascioferro S. (2019). Synthetic small molecules as anti-biofilm agents in the struggle against antibiotic resistance. Eur. J. Med. Chem..

[5] Thappeta K.R.V., Vikhe Y.S., Yong A.M.H., Chan-Park M.B., Kline K.A. (2020). Combined Efficacy of an Antimicrobial Cationic Peptide Polymer with Conventional Antibiotics to Combat Multidrug-Resistant Pathogens. ACS Infect. Dis..

[6] Parrino B., Carbone D., Cirrincione G., Diana P., Cascioferro S. (2020). Inhibitors of antibiotic resistance mechanisms: Clinical applications and future perspectives. Future Med. Chem..

[7] Ericson J.E., Popoola V.O., Smith P.B., Benjamin D.K., Fowler V.G., Benjamin D.K., Clark R.H., Milstone A.M. (2015). Burden of Invasive Staphylococcus Aureus Infections in Hospitalized Infants. JAMA Pediatr..

[8] Defres S., Marwick C., Nathwani D. (2009). MRSA as a cause of lung infection including airway infection, community-acquired pneumonia and hospital-acquired pneumonia. Eur. Respir. J..

[9] Ippolito G., Leone S., Lauria F.N., Nicastri E., Wenzel R.P. (2010). Methicillin-resistant *Staphylococcus aureus*: The superbug. Int. J. Infect. Dis..

[10] Boyle-Vavra S., Daum R.S. (2007). Community-acquired methicillin-resistant *Staphylococcus aureus*: The role of Panton–Valentine leukocidin. Lab. Investig..

[11] Zetola N., Francis J.S., Nuermberger E.L., Bishai W.R. (2005). Community-acquired meticillin-resistant *Staphylococcus aureus*: An emerging threat. Lancet Infect. Dis..

[12] Cascioferro S., Carbone D., Parrino B., Pecoraro C., Giovannetti E., Cirrincione G., Diana P. (2020). Therapeutic strategies to counteract antibiotic resistance in MRSA biofilm-associated infections. ChemMedChem.

[13] Pallen M.J., Lam A.C., Antonio M., Dunbar K. (2001). An embarrassment of sortases—A richness of substrates?. Trends Microbiol..

[14] Maresso A.W., Schneewind O. (2008). Sortase as a Target of Anti-Infective Therapy. Pharmacol. Rev..

[15] Schneewind O., Missiakas D.M. (2012). Protein secretion and surface display in Gram-positive bacteria. Philos. Trans. R. Soc. Lond. B Biol. Sci..

[16] Spirig T., Weiner E.M., Clubb R.T. (2011). Sortase enzymes in Gram-positive bacteria. Mol. Microbiol..

[17] Clancy K.W., Melvin J.A., McCafferty D.G. (2010). Sortase transpeptidases: Insights into mechanism, substrate specificity, and inhibition. Biopolymers.

[18] Foster T.J., Geoghegan J.A., Ganesh V.K., Höök M. (2014). Adhesion, invasion and evasion: The many functions of the surface proteins of *Staphylococcus aureus*. Nat. Rev. Microbiol..

[19] Jonsson I.-M., Mazmanian S.K., Schneewind O., Verdrengh M., Bremell T., Tarkowski A. (2002). On the Role of *Staphylococcus aureus* Sortase and Sortase-Catalyzed Surface Protein Anchoring in Murine Septic Arthritis. J. Infect. Dis..

[20] Bradshaw W.J., Davies A.H., Chambers C.J., Roberts A.K., Shone C.C., Acharya K.R. (2015). Molecular features of the sortase enzyme family. FEBS J..

[21] Mazmanian S.K., Liu G., Jensen E.R., Lenoy E., Schneewind O. (2000). *Staphylococcus aureus* sortase mutants defective in the display of surface proteins and in the pathogenesis of animal infections. Proc. Natl. Acad. Sci. USA.

[22] Oh K.-B., Oh M.-N., Kim J.-G., Shin D.-S., Shin J. (2006). Inhibition of sortase-mediated *Staphylococcus aureus* adhesion to fibronectin via fibronectin-binding protein by sortase inhibitors. Appl. Microbiol. Biotechnol..

[23] Otto M. (2008). Staphylococcal biofilms. Curr. Top. Microbiol. Immunol..

[24] Suree N., Liew C.K., Villareal V.A., Thieu W., Fadeev E.A., Clemens J.J., Jung M.E., Clubb R.T. (2009). The structure of the *Staphylococcus aureus* sortase-substrate complex reveals how the universally conserved LPXTG sorting signal is recognized. J. Biol. Chem..

[25] Cascioferro S., Totsika M., Schillaci D. (2014). Sortase A: An ideal target for anti-virulence drug development. Microb. Pathog..

[26] Suree N., Jung M.E., Clubb R.T. (2007). Recent Advances Towards New Anti-Infective Agents that Inhibit Cell Surface Protein Anchoring in *Staphylococcus aureus* and Other Gram-Positive Pathogens. Mini-Rev. Med. Chem..

[27] Mühlen S., Dersch P., Stadler M., Dersch P. (2016). Anti-virulence Strategies to Target Bacterial Infections. How to Overcome the Antibiotic Crisis: Facts, Challenges, Technologies and Future Perspectives.

[28] Silva L.N., Zimmer K.R., Macedo A.J., Trentin D.S. (2016). Plant Natural Products Targeting Bacterial Virulence Factors. Chem. Rev..

[29] Ng S.B., Kanagasundaram Y., Fan H., Arumugam P., Eisenhaber B., Eisenhaber F. (2018). The 160K Natural Organism Library, a unique resource for natural products research. Nat. Biotechnol..

[30] Irwin J.J., Shoichet B.K. (2016). Docking Screens for Novel Ligands Conferring New Biology. J. Med. Chem..

[31] Zong Y., Bice T.W., Ton-That H., Schneewind O., Narayana S.V.L. (2004). Crystal structures of *Staphylococcus aureus* sortase A and its substrate complex. J. Biol. Chem..

[32] DeLano W.L. (2011). The PyMOL Molecular Graphics System.

[33] Donlan R.M., Costerton J.W. (2002). Biofilms: Survival mechanisms of clinically relevant microorganisms. Clin. Microbiol. Rev..

[34] Cerca N., Martins S., Cerca F., Jefferson K.K., Pier G.B., Oliveira R., Azeredo J. (2005). Comparative assessment of antibiotic susceptibility of coagulase-negative staphylococci in biofilm versus planktonic culture as assessed by bacterial enumeration or rapid XTT colorimetry. J. Antimicrob. Chemother..

[35] Høiby N., Bjarnsholt T., Givskov M., Molin S., Ciofu O. (2010). Antibiotic resistance of bacterial biofilms. Int. J. Antimicrob. Agents.

[36] Roche F.M., Massey R., Peacock S.J., Day N.P., Visai L., Speziale P., Lam A., Pallen M., Foster T.J. (2003). Characterization of novel LPXTG-containing proteins of *Staphylococcus aureus* identified from genome sequences. Microbiology.

[37] DeDent A., Bae T., Missiakas D.M., Schneewind O. (2008). Signal peptides direct surface proteins to two distinct envelope locations of *Staphylococcus aureus*. EMBO J..

[38] Otto M. (2012). MRSA virulence and spread. Cell. Microbiol..

[39] Welsh K.J., Abbott A.N., Lewis E.M., Gardiner J.M., Kruzel M.C., Lewis C.T., Mohr J.F., Wanger A., Armitige L.Y. (2010). Clinical characteristics, outcomes, and microbiologic features associated with methicillin-resistant *Staphylococcus aureus* bacteremia in pediatric patients treated with vancomycin. J. Clin. Microbiol..

[40] Rasko D.A., Sperandio V. (2010). Anti-virulence strategies to combat bacteria-mediated disease. Nat. Rev. Drug Discov..

[41] Rollinger J.M., Stuppner H., Langer T., Petersen F., Amstutz R. (2008). Virtual screening for the discovery of bioactive natural products. Natural Compounds as Drugs Volume I.

[42] Pereira F., Aires-de-Sousa J. (2018). Computational Methodologies in the Exploration of Marine Natural Product Leads. Mar. Drugs.

[43] Parker J.C., McPherson R.K., Andrews K.M., Levy C.B., Dubins J.S., Chin J.E., Perry P.V., Hulin B., Perry D.A., Inagaki T. (2000). Effects of skyrin, a receptor-selective glucagon antagonist, in rat and human hepatocytes. Diabetes.

[44] Jahn L., Schafhauser T., Wibberg D., Rückert C., Winkler A., Kulik A., Weber T., Flor L., van Pée K.-H., Kalinowski J. (2017). Linking secondary metabolites to biosynthesis genes in the fungal endophyte Cyanodermella asteris: The anti-cancer bisanthraquinone skyrin. J. Biotechnol..

[45] Cascioferro S., Raimondi M.V., Cusimano M.G., Raffa D., Maggio B., Daidone G., Schillaci D. (2015). Pharmaceutical Potential of Synthetic and Natural Pyrrolomycins. Molecules.

[46] Raimondi M.V., Listro R., Cusimano M.G., La Franca M., Faddetta T., Gallo G., Schillaci D., Collina S., Leonchiks A., Barone G. (2019). Pyrrolomycins as antimicrobial agents. Microwave-assisted organic synthesis and insights into their antimicrobial mechanism of action. Bioorg. Med. Chem..

[47] Schillaci D., Petruso S., Raimondi M.V., Cusimano M.G., Cascioferro S., Scalisi M., La Giglia M.A., Vitale M. (2010). Pyrrolomycins as potential anti-staphylococcal biofilms agents. Biofouling.

[48] Nitulescu G., Nicorescu I.M., Olaru O.T., Ungurianu A., Mihai D.P., Zanfirescu A., Nitulescu G.M., Margina D. (2017). Molecular Docking and Screening Studies of New Natural Sortase A Inhibitors. Int. J. Mol. Sci..

[49] Oniga S.D., Araniciu C., Palage M.D., Popa M., Chifiriuc M.-C., Marc G., Pirnau A., Stoica C.I., Lagoudis I., Dragoumis T. (2017). New 2-Phenylthiazoles as Potential Sortase A Inhibitors: Synthesis, Biological Evaluation and Molecular Docking. Molecules.

[50] Kahlon A.K., Negi A.S., Kumari R., Srivastava K.K., Kumar S., Darokar M.P., Sharma A. (2014). Identification of 1-chloro-2-formyl indenes and tetralenes as novel antistaphylococcal agents exhibiting sortase A inhibition. Appl. Microbiol. Biotechnol..

[51] Suree N., Yi S.W., Thieu W., Marohn M., Damoiseaux R., Chan A., Jung M.E., Clubb R.T. (2009). Discovery and Structure-Activity Relationship Analysis of *Staphylococcus aureus* Sortase A Inhibitors. Bioorg. Med. Chem..

[52] Irwin J.J., Shoichet B.K., Mysinger M.M., Huang N., Colizzi F., Wassam P., Cao Y. (2009). Automated docking screens: A feasibility study. J. Med. Chem..

[53] Krivov G.G., Shapovalov M.V., Dunbrack R.L. (2009). Improved prediction of protein side-chain conformations with SCWRL4. Proteins.

[54] Mysinger M.M., Shoichet B.K. (2010). Rapid Context-Dependent Ligand Desolvation in Molecular Docking. J. Chem. Inf. Model..

[55] O’Toole G.A. (2011). Microtiter dish biofilm formation assay. J. Vis. Exp..

[56] Torres N.S., Abercrombie J.J., Srinivasan A., Lopez-Ribot J.L., Ramasubramanian A.K., Leung K.P. (2016). Screening a commercial library of pharmacologically active small molecules against *Staphylococcus aureus* biofilms. Antimicrob. Agents Chemother..

[57] Elgalai I., Foster H.A. (2003). Comparison of adhesion of wound isolates of *Staphylococcus aureus* to immobilized proteins. J. Appl. Microbiol..

