# Supplementary material for: In-Silico Identified New Natural Sortase A Inhibitors Disrupt S. aureus Biofilm Formation
Source: Int J Mol Sci. 2020 Nov 14;21(22):8601. doi: 10.3390/ijms21228601 (PMC7696255; doi:10.3390/ijms21228601)
Supplement: Supplementary file 1 [file ijms-21-08601-s001.pdf]

In-silico identified new natural sortase A inhibitors disrupt *S. aureus* biofilm formation

Kishore Reddy Venkata Thappeta[1#], Li Na Zhao[2#,3], Choy Eng Nge [1], Sharon Crasta[1], Chung Yan Leong [1], Veronica Ng[1], Yoganathan Kanagasundaram[1,2*], Hao Fan[2,*] and Siew Bee Ng [1,2*]

[1]Singapore Institute of Food and Biotechnology Innovation (SIFBI), Agency for Science, Technology and Research (A*STAR), 31 Biopolis Way, #01-02 Nanos, Singapore 138669

[2]Bioinformatics Institute (BII), Agency for Science, Technology and Research (A*STAR), 30 Biopolis Street, #07-01 Matrix, Singapore 138671

[3]Institute of Molecular and Cell Biology (IMCB), Agency for Science, Technology and Research (A*STAR), 61 Biopolis Drive, #3-09 Proteos, Singapore 138673

[#]These two authors contributed equally to this work

*To whom correspondence should be addressed: Ng Siew Bee, Singapore Institute of Food and Biotechnology Innovation (SIFBI), Agency for Science, Technology and Research (A*STAR), 31 Biopolis Way, #01-02 Nanos, Singapore 138669, Tel.: 65-6478 8513, E-mail: ngsb@sifbi.a-star.edu.sg; Hao Fan, Bioinformatics Institute (BII), Agency for Science, Technology and Research (A*STAR), 30 Biopolis Street, #07-01 Matrix, Singapore 138671, Tel.: 65-6478 8500, E-mail: fanh@bii.a-star.edu.sg; Yoganathan Kanagasundaram, Singapore Institute of Food and Biotechnology Innovation (SIFBI), Agency for Science, Technology and Research (A*STAR), 31 Biopolis Way, #01-02 Nanos, Singapore 138669, Tel.: 65-6586 9508, E-mail: yoganathank@sifbi.a-star.edu.sg

Supplementary Materials:

Figure S1: Dose response of known inhibitors, curcumin (A) and chlorogenic acid (B), on sortase A. Inhibition of SrtA activity was measured at 24h using two SrtA substrates. The results are mean values and SE of three replicates.

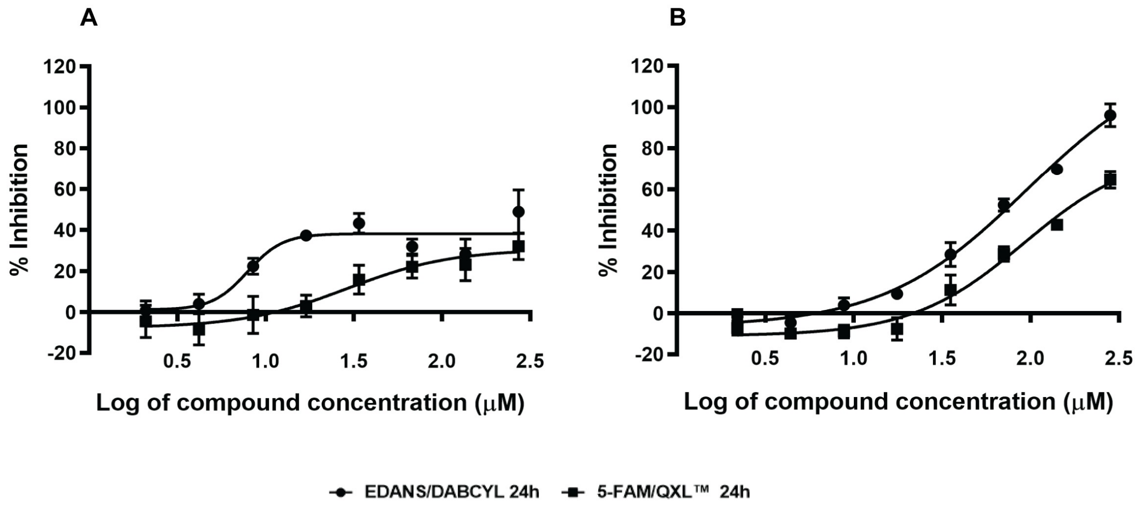

Figure S2: Effect of hit compounds and known inhibitors on the growth of *S. aureus* ATCC 25923: N1287 (A), N2576 (B), curcumin (C), and chlorogenic acid (D). The results are mean values and SE of three replicates.

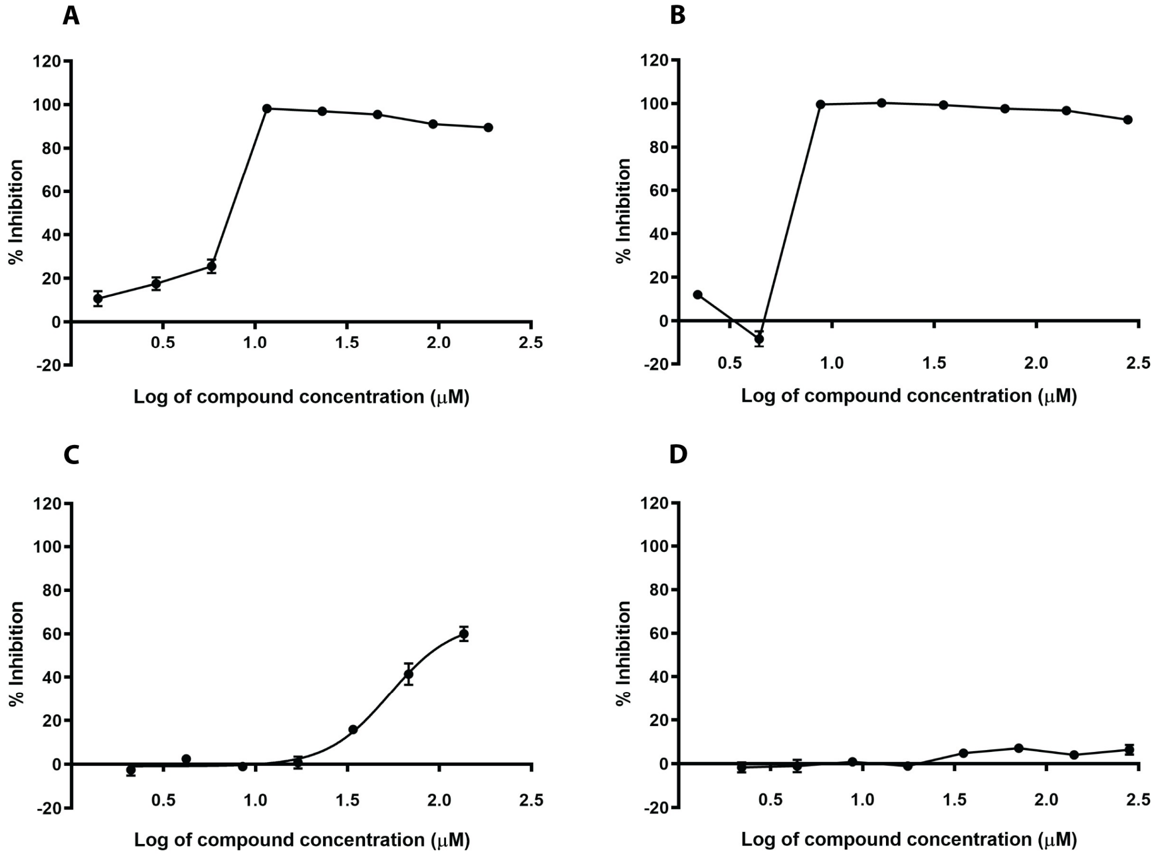

Figure S3: Effect of hit compounds and known inhibitors on the growth of *S. aureus* ATCC 33591 (MRSA): N1287 (A), N2576 (B), curcumin (C), and p-HMB (D). The results are mean values and SE of three replicates.

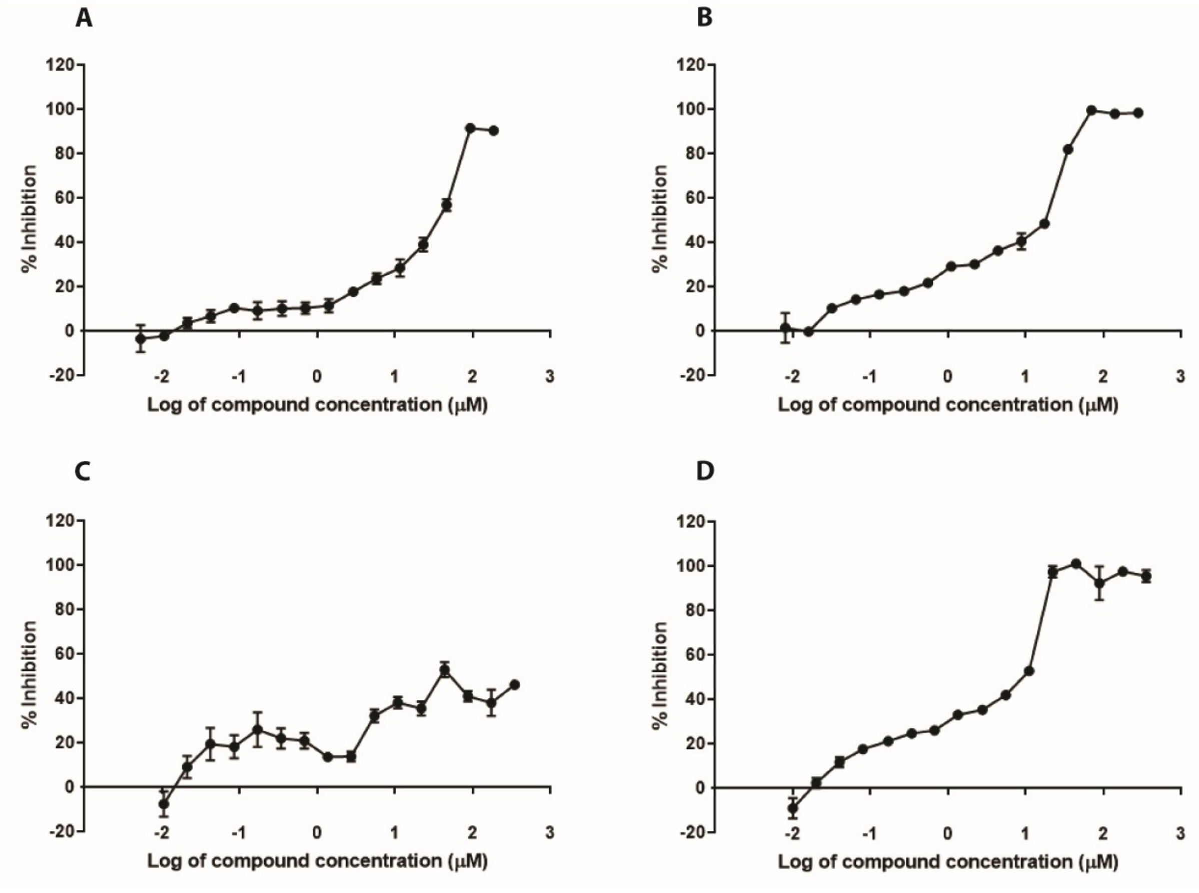

Figure S4. Effect of the known inhibitors of SrtA and selected hits on *S. aureus* ATCC 33591 (MRSA) biofilm formation and pre-formed biofilms. Inhibition of *S. aureus* biofilm formation was determined using CV assay. N1287 (A), N2576 (B), curcumin (C) and p-HMB (D). The results data are presented as the mean percent inhibition ± S.E of three replicates (two independent experiments) relative to the untreated control (wild type). *p<0.05, **p<0.01, ***p<0.001 and, ****p<0.0001.

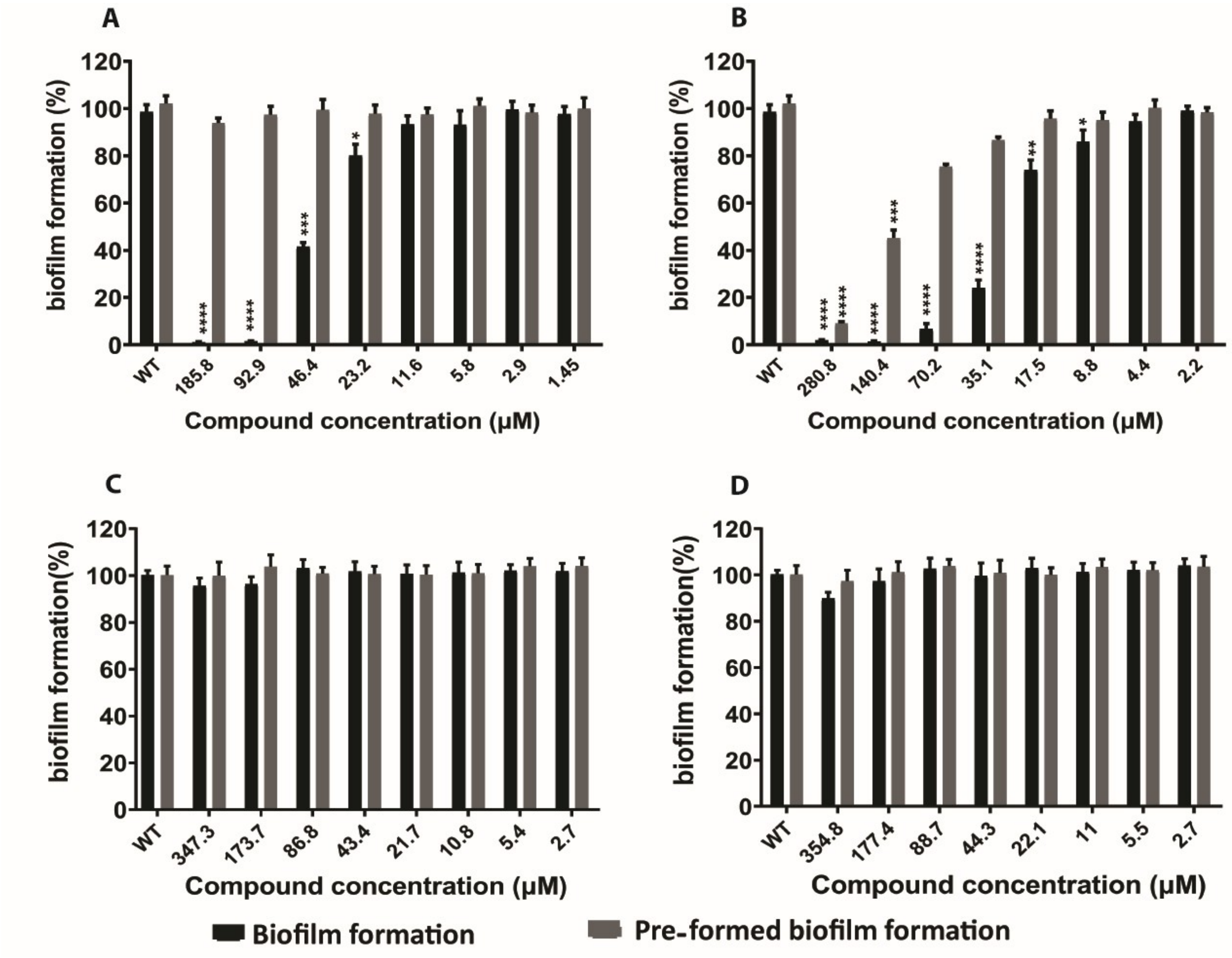

Figure S5: Crystal violet assay to assess the antibiofilm activity of selected hits and known inhibitors of SrtA on *S. aureus* ATCC 33591 (MRSA) biofilm formation (A) and pre-formed biofilms (B).

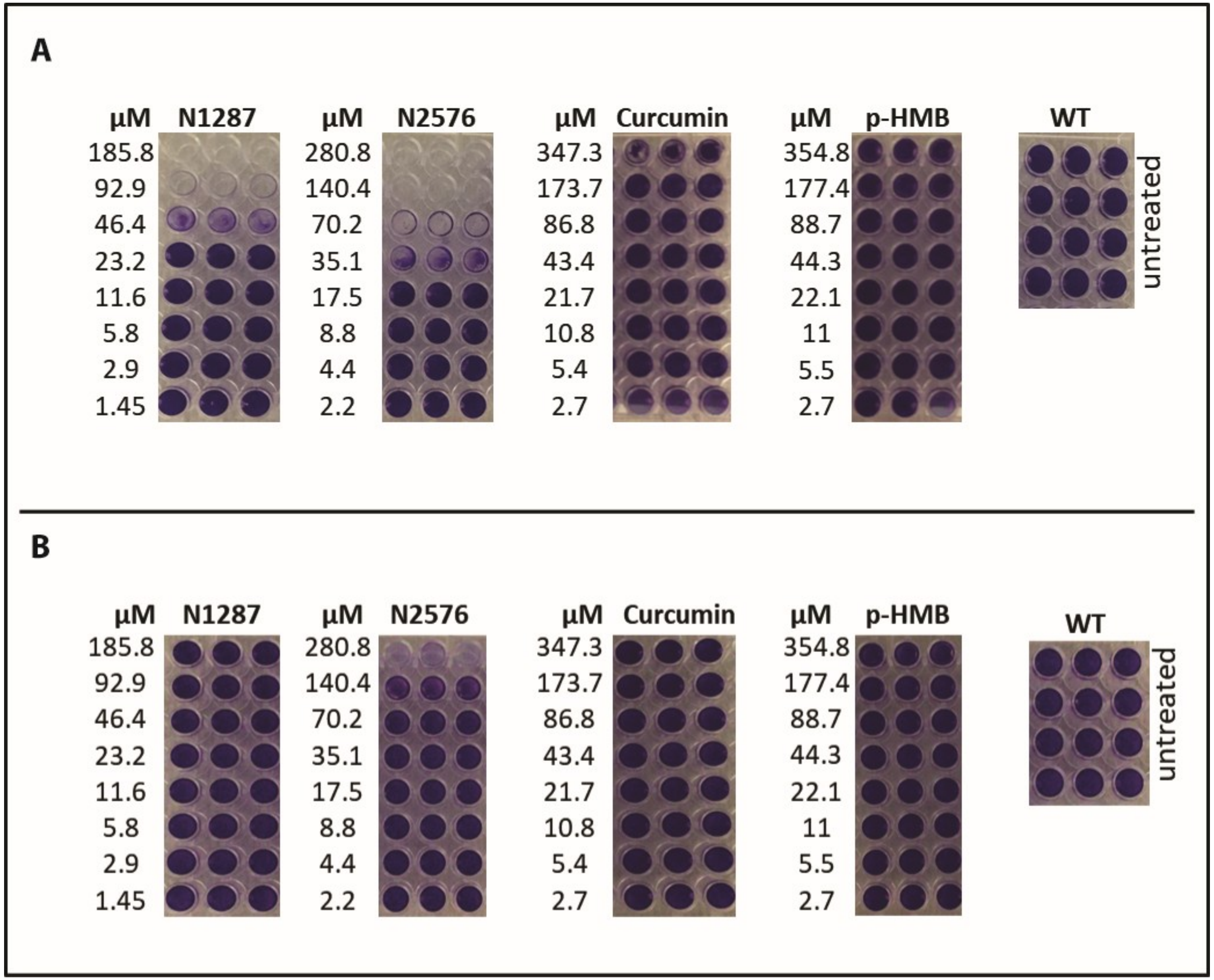

Figure S6: Dose-response of the known inhibitors of SrtA and selected hits on *S. aureus* ATCC 33591 (MRSA) biofilm formation and pre-formed biofilms. Inhibition of *S. aureus* biofilm was determined using PrestoBlue cell viability reagent. N1287 (A), N2576 (B), curcumin (C), and p-HMB (D). The results data

are presented as the mean percent inhibition ± S.E of three replicates (two independent experiments) relative to the untreated control (wild type). *p<0.05, **p<0.01, ***p<0.001 and, ****p<0.0001.

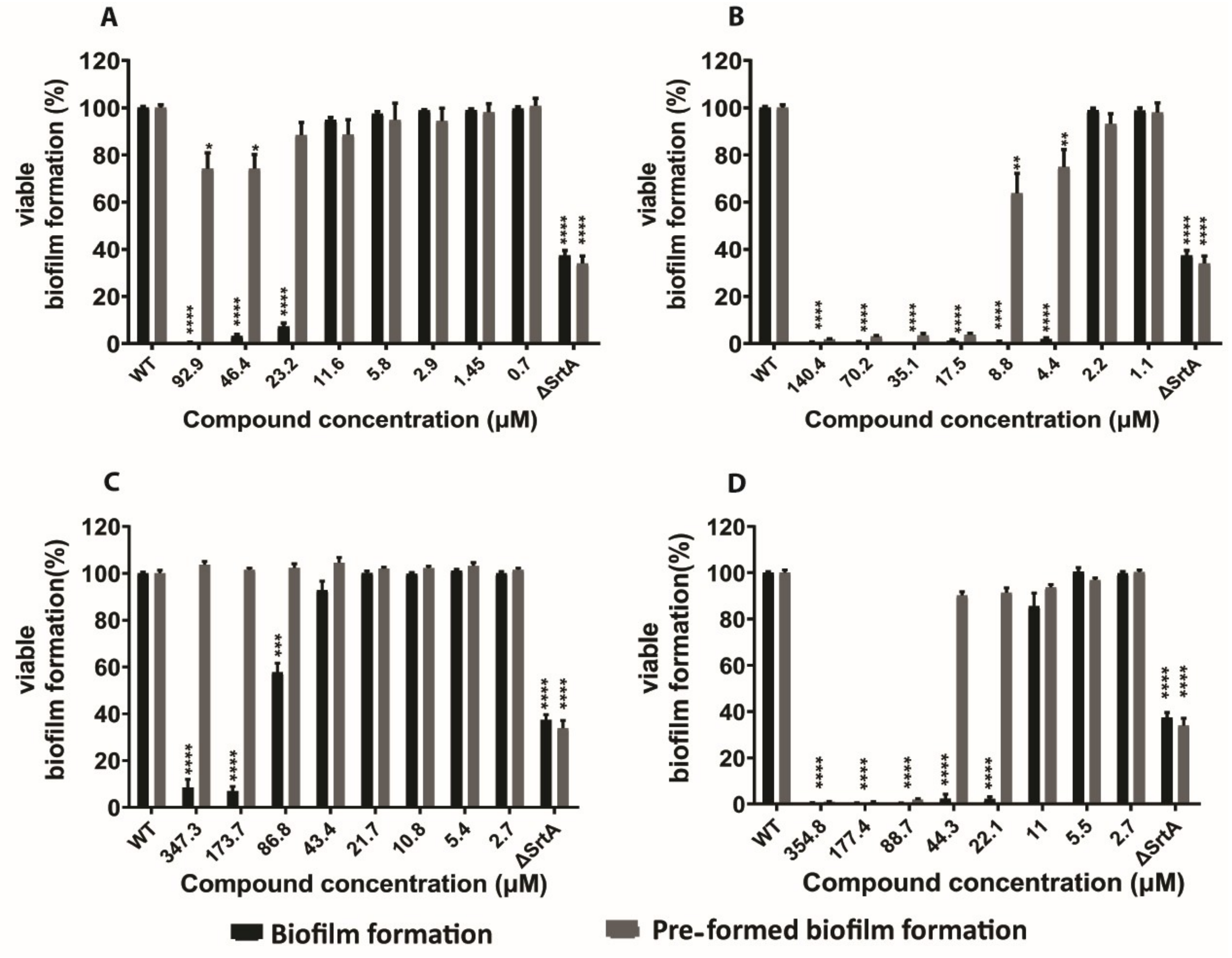

Figure S7: Effect of the known inhibitors of SrtA and selected hits on *E.coli* ATCC 25922 biofilm formation. Inhibition of *E.coli* ATCC 25922 biofilm formation was determined using CV assay. N1287 (A), N2576 (B), curcumin (C) and p-HMB (D). The results data are presented as the mean percent biofilm formation ± S.E of three replicates (two independent experiments) relative to the untreated control (wild type).

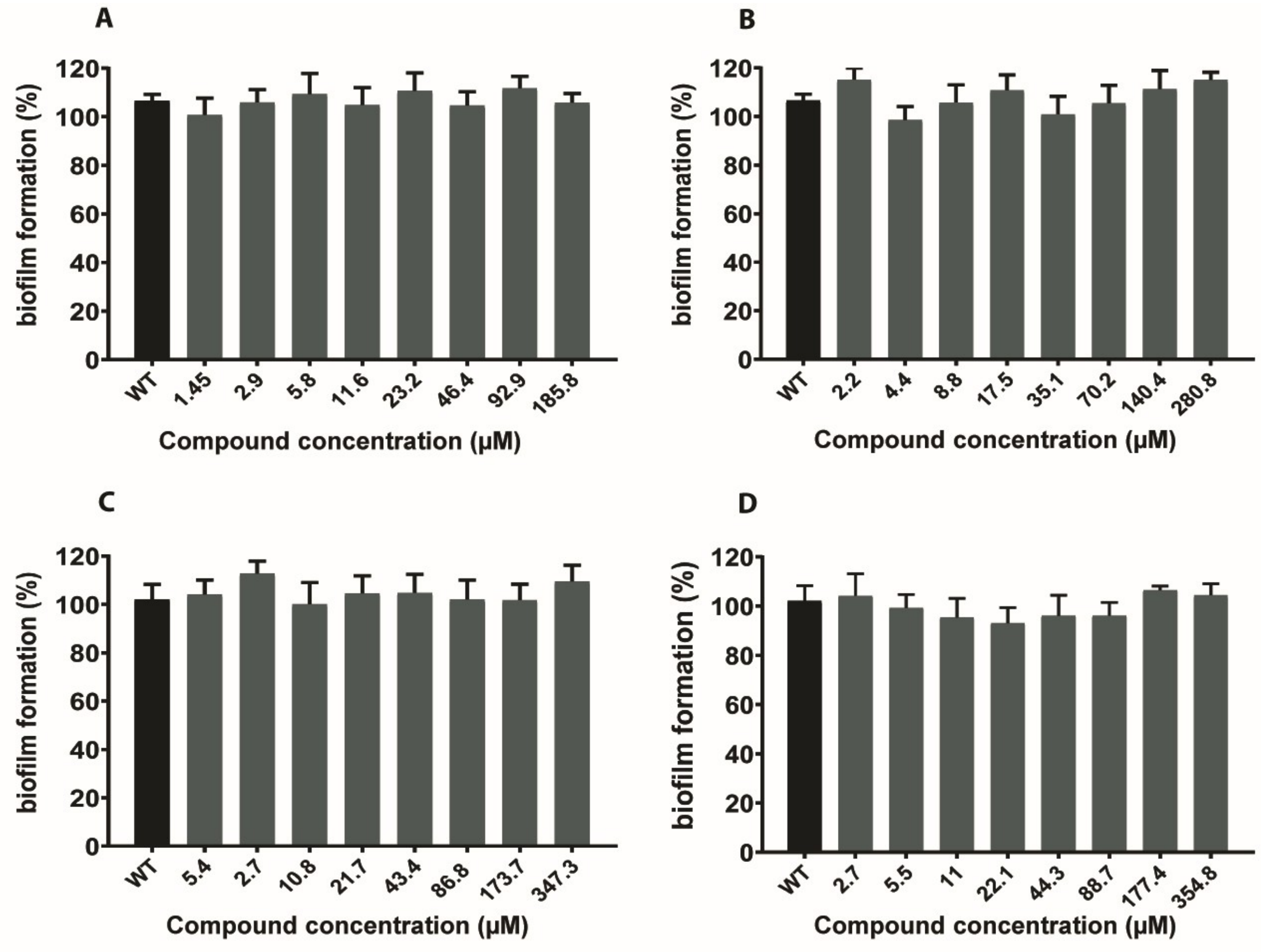

Table S1: Biological activity of selected hits and standard inhibitors

Compound	*S. aureus* MIC (μM)			Mammalian cell lines IC_{50} (μM)		Biofilm Inhibition IC_{50} (μM) *S. aureus* Newman		Biofilm Inhibition IC_{50} (μM) *S. aureus* ATCC 33591 (MRSA)		Fibrinogen adherence Inhibition (IC_{50}, μM)
	ATCC 25923	Newman	ATCC 33591 (MRSA)	HepG2	A549	Biofilm formation	Pre-formed	Biofilm formation	Pre-formed	
N1287	7.2 ± 0.1	7.3±0.8	80.1±6.3	63.9±5.1	64.7±6.2	12.2±4.1	NA	41.9	NA	9.5±0.5
N2576	6.3 ± 0.1	5.8±1.3	25.2±5.5	65±7.4	88.1±13	2.9±0.4	10±1.2	24.8	>200	5.7±0.8
Curcumin	> 200	>200	>200	39.6±5.3	48.2±12.7	51.2±4.4	NA	>150	NA	46.5±6.2
Chlorogenic acid	> 200	>200	NT	>200	>200	NT	NT	NT	NT	NT
p-hydroxymer-curibenzoic acid (p-HMB)	NT	NT	6.7±1.5	NT	NT	11.9±2.8	43±3.6	>150	NA	37.38±5.3

NA –no activity, NT-not tested
